# An LcMYB111-LcHY5 Module Differentially Activates an *LcFLS* Promoter in Different Litchi Cultivars

**DOI:** 10.3390/ijms242316817

**Published:** 2023-11-27

**Authors:** Zhidan Xiao, Jing Wang, Nonghui Jiang, Chao Fan, Xu Xiang, Wei Liu

**Affiliations:** Institute of Fruit Tree Research, Guangdong Academy of Agricultural Sciences, Key Laboratory of South Subtropical Fruit Biology and Genetic Resource Utilization, Ministry of Agriculture and Rural Affairs, Guangdong Provincial Key Laboratory of Tropical and Subtropical Fruit Tree Research, Guangzhou 510640, China; xiaozhidan@gdaas.cn (Z.X.); wanglab12@163.com (J.W.); jiangnonghui@gdaas.cn (N.J.); gdfanchao@163.com (C.F.); xiangxu@vip.163.com (X.X.)

**Keywords:** LcFLS, flower color, flavonol, LcMYB111, LcHY5

## Abstract

Flavonol synthase (FLS) is the crucial enzyme of the flavonol biosynthetic pathways, and its expression is tightly regulated in plants. In our previous study, two alleles of *LcFLS,* *LcFLS-A* and *LcFLS-B*, have been identified in litchi, with extremely early-maturing (EEM) cultivars only harboring *LcFLS-A*, while middle-to-late-maturing (MLM) cultivars only harbor *LcFLS-B*. Here, we overexpressed both *LcFLS* alleles in tobacco, and transgenic tobacco produced lighter-pink flowers and showed increased flavonol levels while it decreased anthocyanin levels compared to WT. Two allelic promoters of *LcFLS* were identified, with EEM cultivars only harboring proLcFLS-A, while MLM cultivars only harbor proLcFLS-B. One positive and three negative R2R3-MYB transcription regulators of *LcFLS* expression were identified, among which only positive regulator *LcMYB111* showed a consistent expression pattern with *LcFLS*, which both have higher expression in EEM than that of MLM cultivars. LcMYB111 were further confirmed to specifically activate proLcFLS-A with MYB-binding element (MBE) while being unable to activate proLcFLS-B with mutated MBE (MBEm). LcHY5 were also identified and can interact with LcMYB111 to promote *LcFLS* expression. Our study elucidates the function of *LcFLS* and its differential regulation in different litchi cultivars for the first time.

## 1. Introduction

Flavonoids are a major group of plant-specialized metabolites [1,2]. Moreover, flavonols are the most abundant flavonoid compounds in plants and have received much attention due to multifaceted physiological functions, including plant growth, development, reproduction, and responses to biotic and abiotic stress [3,4,5,6]. Many *FLS* genes have been identified in plants [7,8,9,10,11], and their enzymatic properties and functionalities were characterized, such as AtFLS1, CitFLS, GbFLS, and OsFLS, which are bifunctional enzymes exhibiting both FLS and flavanone 3-hydroxylase (F3H) activity.

Flavonols are pivotal regulators of the pigmentation of flowers [12]. In the red and white flower phenotypes of *Rosa rugosa*, *Rosa multiflora*, *Prunus persica*, *Dianthus caryophyllus*, *Rhododendron simsii*, *Camellia japonica*, and *Petunia hybrida*, flavonols could be detected in red and white flowers, but anthocyanins were almost undetectable in the white cultivar [13]. Dihydrokaempferol and dihydroquercetin are two important precursors of flavonols and anthocyanins synthesis, which could be catalyzed either by flavonol synthase (*FLS*) to form copigment flavonols (kaempferol and quercetin) [14], or by dihydroflavonol-4-reductase (DFR) to form leucoanthocyanidins in the anthocyanin biosynthesis pathway [15]. Overexpressing *FLS* genes from *P. hybrida*, *R. rugosa*, and *Allium cepa* L. in tobacco lead to flowers with a lighter or white color which contain increased levels of flavonol and decreased levels of anthocyanin [16,17,18], while silencing *FLS* genes caused a decrease in flavonol contents and an increase in anthocyanin levels, resulting in flowers with enhanced coloration [19,20,21].

It is known that the R2R3-MYB family genes are important transcriptional regulators of flavonoid biosynthesis [22,23]. One hundred twenty-six R2R3-MYB proteins have been divided into 23 subgroups in *Arabidopsis thaliana* [24]. All R2R3-MYB family members contain R2 and R3 highly conserved imperfect repeats at the N-terminus, while the C-terminus is highly diverse [25,26,27]. Most R2R3-MYB transcription factors regulate flavonol synthesis via combinations and interactions with cofactors WDR and bHLH proteins [28,29,30]. Notably, in the control of the flavonol biosynthetic pathway, three members of subgroup 7 (SG7) of the R2R3-MYB family (AtMYB12/PFG1, AtMYB11/PFG2, and AtMYB111/PFG3) were first identified in *A. thaliana*, acting independently of bHLH cofactors [31,32,33,34], which individually activate a group of flavonol biosynthetic genes, including *Chalcone synthase (CHS)*, *Chalcone isomerase* (*CHI*), *F3H*, and *FLS* [35,36,37,38,39]. However, *AtMYB4* and *AtMYB7* of subgroup 4 (SG4) have been demonstrated as transcriptional repressors in the control of the flavonol biosynthetic pathway [40]. R2R3-MYB activators and repressors are forming a regulatory network together to fine-tune flavonol biosynthesis in plants [41,42].

The bZIP transcription factor HY5 (ELONGATED HYPOCOTYL 5) inhibits hypocotyl growth and lateral root development and promotes pigment accumulation in a light-dependent manner in *Arabidopsis* [43]. HY5 directly binds to the G-box and ACE motifs in the promoters of anthocyanin biosynthesis genes such as *CHS*, *CHI*, and *FLS*, as well as transcription factors like *MYB111* and *MYB12*, thereby regulating anthocyanin biosynthesis in *Arabidopsis* [44]. SlHY5 regulates tomato anthocyanin biosynthesis by binding to the G-box and ACE motifs in the promoters of *CHS* and *DFR* [45]. HY5 in *Malus* × *domestica* (MdHY5) can also bind to G-box elements in the promoter region of the *MdMYB10* gene regulating light-induced anthocyanin biosynthesis [46]. PyHY5 recognizes and binds G-box elements in PyMYB10 promoters, enhances their activity, and promotes the accumulation of anthocyanins in red pear peel [47]. HY5 is a positive regulator of anthocyanin accumulation through direct binding to the promoters of genes involved in anthocyanin biosynthesis.

Our previous study has found that *LcFLS* (LITCHI007338.m1) expression levels and flavonol contents were much higher in extremely early-maturing (EEM) cultivars than those in middle-to-late-maturing (MLM) cultivars in litchi [48]. In this study, we aimed to further elucidate the function of *LcFLS* and the regulatory mechanism of its expression in different litchi cultivars. Using a range of techniques, the FLS enzyme activity and the mode of regulation of the *FLS* gene in different litchi cultivars were established.

## 2. Results

### 2.1. Transgenic Tobacco Expressing LcFLS Has Lighter-Pink Flowers and Increased Flavonol Levels Than the Wild-Type

Two different alleles of the *LcFLS* gene, *LcFLS-A* and *LcFLS-B*, have been identified in litchi cultivars in our previous study, with EEM cultivars only harboring *LcFLS-A* allele, while MLM cultivars only harbor *LcFLS-B* allele [48]. To investigate the biological function of *LcFLS* in litchi, we first examined the flavonol synthetase activity of LcFLS proteins. We constructed recombinant LcFLS-A and -B proteins fused with Trx (thioredoxin) tag (Trx-LcFLS-A and Trx-LcFLS-B) and then expressed in *E. coli* to determine whether they harbored FLS activity. After the induction of expression with IPTG, SDS–PAGE confirmed that recombinant proteins were successfully expressed in the cultures (Figure S1A), and most of the Trx-LcFLS protein was in the soluble fraction. The FLS activity of the purified recombinant proteins was assayed using dihydroquercetin (DHQ) and dihydrokaempferol (DHK) as substrates in the presence of ferrous iron and 2-oxoglutarate. HPLC analysis showed that recombinant LcFLS-A and -B proteins catalyzed the formation of kaempferol and quercetin from dihydrokaempferol and dihydroquercetin (Figure S1B), respectively. These results indicate that both LcFLS-A and -B proteins exhibit FLS activity.

**Figure 1:**
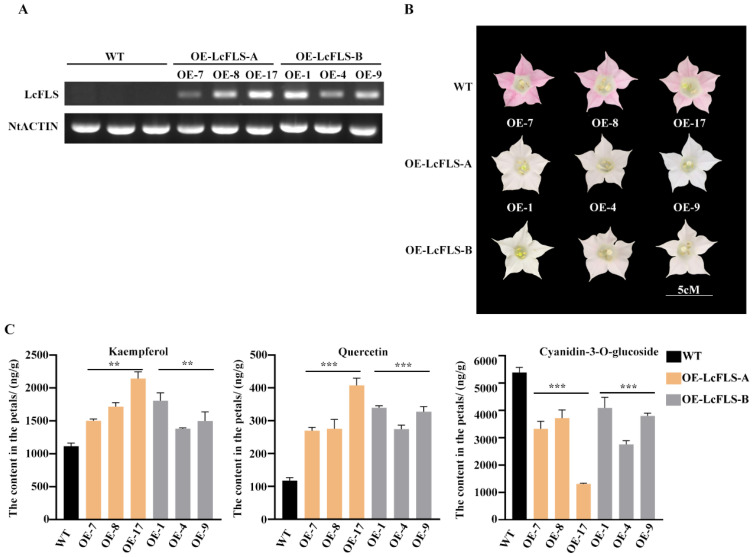
Phenotypes of flowers and flavonoid contents in the petals of transgenic tobacco overexpressing *LcFLS*. (**A**) Expression analysis of *LcFLS* in the flowers of wild-type and transgenic tobacco by semiquantitative RT-qPCR. The *NtACTIN* gene was set up as an inner control. (**B**) Flower phenotypes of wild-type and transgenic tobacco overexpressing *LcFLS-A* and *LcFLS-B*. WT, K326 wild-type; the OE-7, OE-8, OE-17 represent three different tobacco lines overexpressing *LcFLS-A* (OE-LcFLS-A); OE-1, OE-4, OE-9 represent three different tobacco lines overexpressing *LcFLS-B* (OE-LcFLS-B). (**C**) Flavonoid and anthocyanin contents in the petals of wild-type and transgenic tobacco overexpressing *LcFLS-A* and *LcFLS-B*. The error bars indicate ±SD from three replicates. Statistical significance was determined by one-way ANOVA with Tukey’s tests. The asterisks indicate that the value is significantly different from that of the WT (** *p* < 0.01, *** *p* < 0.001).

Both *LcFLS* alleles were overexpressed in tobacco, using the CaMV 35S promoter. Six independent transgenic lines of tobacco overexpressing *LcFLS* were successfully isolated from Hyg resistance selection, and their progenies (T3 generation) were generated (*LcFLS-A OE-7,8,17* and *LcFLS-B OE-1,4,9*). We examined *LcFLS* transcript abundance in transgenic tobacco petals by semiquantitative RT-PCR (qPCR). As can be seen from the result of agarose gel electrophoresis, the *LcFLS* gene were highly expressed in transgenic petals, but no bands were amplified in wild-type (WT) tobacco (Figure 1A). Compared with the pink flower of the WT, the flower color of *OE-LcFLS-A* and *OE-LcFLS-B* transgenic tobacco becomes lighter and almost white; however, there is no significant difference between them (Figure 1B).

For further verification of the function of *LcFLS* in flavonoid biosynthesis, the expression levels of flavonoid pathway genes in WT and LcFLS-A and-B overexpressing transgenic lines were determined by qRT-PCR. The results showed that the expression levels of *NtCHI*, *NtANS*, *NtLAR*, and *NtUFGT* in anthocyanins biosynthesis were somewhat increased in transgenic lines, while those of *NtFLS* decreased by approximately 60% in transgenic lines. It may be the result of negative feedback regulation due to *LcFLS* overexpression (Figure S2). To further verify the function of *LcFLS* in flavonoid biosynthesis, flavonols and anthocyanins were extracted from *LcFLS* transgenic tobacco petals, and their contents were analyzed using high-performance liquid chromatography (HPLC) analysis. The contents of kaempferol and quercetin, two major types of flavonols, were increased 1.2–1.9 times and 2.3–3.4 times, respectively, in transgenic petals compared to the WT (Figure 1C). The contents of cyanidin-3-O-glucoside, the major class of anthocyanins in the petals of tobacco flowers, were greatly reduced in transgenic petals relative to the WT (Figure 1C).

The results indicated that *LcFLS* is a key enzyme regulating the synthesis of flavonoids, with the functional overexpression of *LcFLS* in tobacco causing increased flavonol accumulation and decreased anthocyanin accumulation.

### 2.2. Characterization of Four Candidate R2R3-MYB Transcriptional Factors and Two Allelic proLcFLS of Litchi

**Figure 2:**
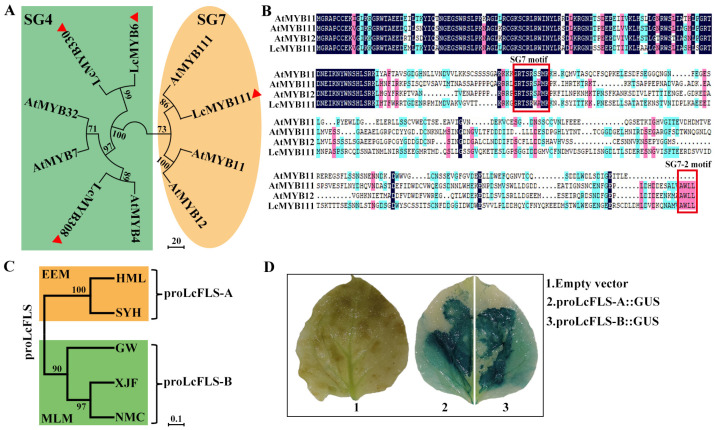
Phylogenetic analysis of four candidate LcMYB transcriptional factors and two allelic proLcFLS. (**A**) Phylogenetic tree of SG4 and SG7 R2R3-MYB family members in litchi and *Arabidopsis*. The phylogenetic tree was constructed using the neighbor-joining method in the MEGA-X software with 1000 bootstrap replicates. The numbers at the nodes indicate the bootstrap values, with cutoff no less than 50. (**B**) Sequence alignment of LcMYB111 with known flavonol regulators AtMYB11, AtMYB12, and AtMYB111. The alignment was generated using the DNAMAN 6.0 software. The R2 and R3 repeats of the MYB domain, as well as S7 motif (GRTxRSxMK) and SG7-2 motif [(W/x)(L/x)LS] which were denoted by red rectangle were found in LcMYB111 sequence. (**C**) The phylogenetic tree of LcFLS promoters cloned from EEM cultivars (‘SYH’ and ‘HML’) and MLM cultivars (‘NMC’, ‘XJF’, and ‘GW’). The phylogenetic tree was generated using the neighbor-joining method in the MEGA-X software with 1000 bootstrap replicates. (**D**) Analysis of GUS activity of proLcFLS-A and proLcFLS-B. 1: Empty vector, 2: proLcFLS-A::GUS, 3: proLcFLS-B::GUS.

We searched for homologous R2R3-MYB genes in the litchi genome (http://www.sapindaceae.com/ (accessed on 1 January 2022)) using three members of SG4 (AtMYB4, AtMYB7, and AtMYB32) and three members of SG7 (AtMYB11, AtMYB12, and AtMYB111), which regulate the synthesis of flavonols in *A. thaliana* as queries. Finally, we obtained four litchi R2R3-MYB homologues, which were named as *LcMYB111*, *LcMYB6*, *LcMYB308*, and *LcMYB330* using the gene annotation information of the litchi genome database. Phylogenetic analysis of the four litchi R2R3-MYB homologues and the abovementioned six *A. thaliana* R2R3-MYB proteins showed that LcMYB111 clustered with SG7, while *LcMYB6*, *LcMYB308*, and *LcMYB330* clustered with SG4 (Figure 2A). The full-length cDNA sequence of the *LcMYB111*, *LcMYB6*, *LcMYB308*, and *LcMYB330* were cloned from the representative EEM cultivar ‘Sanyuehong’ (‘SYH’) and MLM cultivar ‘Nuomici’ (‘NMC’) and sequenced, respectively. The protein sequence of *LcMYB111* and *LcMYB330* of ‘SYH’ and ‘NMC’ were identical, and *LcMYB6* and *LcMYB308* of ‘SYH’ and ‘NMC’ shared 98% and 97% identity, respectively (Figure 2C and Figure S3). As the sequences of the four R2R3-MYB transcription factors were highly conserved between ‘SYH’ and ‘NMC’, the cDNA sequences cloned from ‘SYH’ were used for subsequent experimental analysis. Amino acid alignment of the LcMYB111 and three SG7 protein sequences of *A. thaliana* showed that LcMYB111 contains the two highly conserved R2R3 domains: the typical SG7 motif (GRTxRSxMK) and SG7-2 motifs [(W/x)(L/x)LS] (Figure 2B), indicating its putative function in regulating flavonol biosynthesis in litchi.

We further cloned the 1.8 kb promoter sequences of *LcFLS-A* and *LcFLS-B* from representative EEM cultivars (‘SYH’ and ‘Hemaoli’ (HML)) and MLM cultivars (‘NMC’, ‘Xianjinfeng’ (XJF) and ‘Guiwei’ (GW)), respectively. Sequence alignment and phylogenetic analysis of the obtained sequences showed that *LcFLS* promoters (*proLcFLS*) also have two allelic sequences, which were named as proLcFLS-A and proLcFLS-B, with EEM cultivars only harboring proLcFLS-A, while MLM cultivars only harbor proLcFLS-B (Figure 2C and Figure S4). Sequence alignment of proLcFLS-A and proLcFLS-B showed 88.4% identity (Figure S4). proLcFLS-A and proLcFLS-B were fused with GUS and transformed into tobacco leaves and showed GUS activity after dyeing, indicating that both promoters can initiate gene expression (Figure 2D).

### 2.3. Four R2R3-MYB Transcription Factors Regulate LcFLS Gene Expression and Higher Expression Level in EEM Cultivars

**Figure 3:**
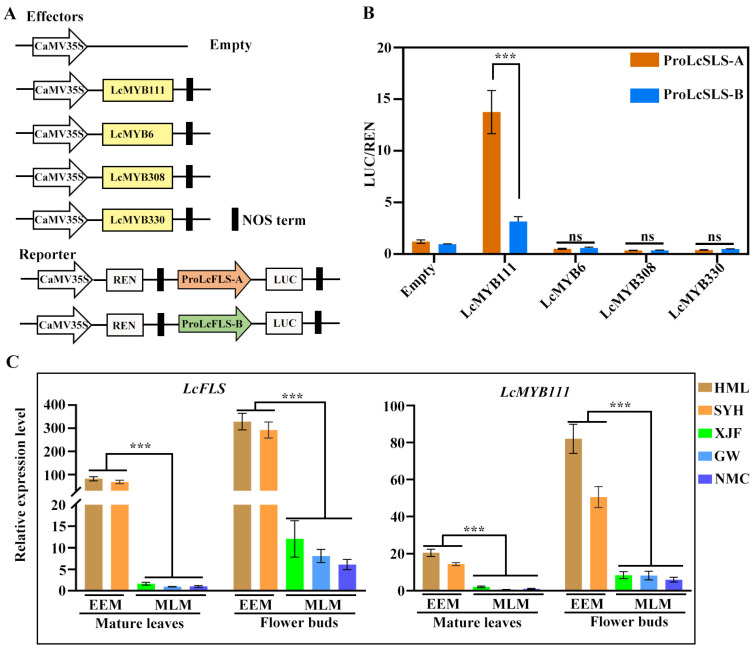
Activity of four R2R3-MYB transcription factors and their expression level in litchi. (**A**) Diagram of constructs used for a transcription activity assay as shown in (**B**). LcMYB111, LcMYB6, LcMYB308, and LcMYB330 were used as effectors, and proLcFLS-A and proLcFLS-B were used as reporters. (**B**) Transcription activity analysis showing the effect of four LcMYBs on the transactivation activity of proLcFLS-A and -B. LUC/REN ratios represent the transcription regulation activities and were normalized to the activity of control. All the bars indicate the mean ± SD of three repeated experiments (*** *p* < 0.001; ns, no significance; one-way ANOVA with Tukey’s tests). (**C**) Expression pattern of *LcFLS* and *LcMYB111* in mature leaves and flower buds of litchi EEM cultivars (‘HML’ and ‘SYH’) and MLM cultivars (‘XJF’, ‘GW’, and ‘NMC’). Relative gene expression levels were calculated as the ratio of ‘HML’, ‘SYH’, ‘XJF’, and ‘GW’ to ‘NMC’, respectively, after being normalized against *LcActin*. The mean ± SD values from three independent experiments are shown. Statistical significance was determined by one-way ANOVA with Tukey’s tests. No significance (ns), asterisks indicated significantly expression difference (*** *p* < 0.001).

To investigate whether these four litchi R2R3-MYB genes have transcriptional regulatory functions on proLcFLS-A and proLcFLS-B, we performed a dual luciferase assay (LUC) on *Arabidopsis* protoplast (Figure 3). LcMYB111, LcMYB6, LcMYB308, and LcMYB330 were used as effectors, while proLcFLS-A and proLcFLS-B were used to drive a *LUC* (Firefly luciferase) reporter gene with *REN* (Renilla luciferase) as a reference gene (Figure 3A). Our results indicated that LcMYB111 had obvious transcriptional activation activity with proLcFLS-A (13.7-fold), whereas weak transcriptional activation activity was observed with proLcFLS-B (2.5-fold) compared with the control (Figure 3B). On the contrary, LcMYB6, LcMYB308, and LcMYB330 could evidently suppress the transcriptional activity of proLcFLS-A and proLcFLS-B, yet there is inconspicuous difference between them (Figure 3B).

To explore the correlation between the expression level of *LcFLS* and the four R2R3-MYB genes in litchi EEM and MLM cultivars, the transcript levels of *LcFLS* and the four R2R3-MYB genes in mature leaves and flower buds of the representative EEM cultivars (‘SYH’ and ‘HML’) and MLM cultivars (‘NMC’, ‘XJF’, and ‘GW’) were analyzed using qRT-PCR. As shown in Figure 3C, the transcript levels of *LcFLS* and *LcMYB111* in mature leaves and flower buds were generally significantly higher in EEM cultivars compared with MLM cultivars. In the mature leaves, the *LcFLS* transcript levels were 82- and 68-fold higher in EEM cultivar ‘HML’ and ‘SYH’, respectively, than in ‘NMC’. In the flower buds, the *LcFLS* transcript levels reached a 53- and 47-fold higher level in ‘HML’ and ‘SYH’, respectively, than in ‘NMC’. Similarly, in the mature leaves, the *LcMYB111* transcript levels were 20- and 14-fold higher in EEM cultivars; in the flower buds, the *LcMYB111* transcript levels were 13- and 8-fold higher in EEM cultivars than in ‘NMC’. However, there is no general difference of the expression pattern of the *LcMYB6*, *LcMYB308*, and *LcMYB330* between EEM and MLM cultivars (Figure S5). Therefore, we carefully speculate that the higher expression of *LcFLS* in EEM cultivars may be regulated by the transcriptional activation of LcMYB111.

### 2.4. LcMYB111 Regulates LcFLS Gene Expression by Directly Binding to Specific MBE of ProLcFLS

**Figure 4:**
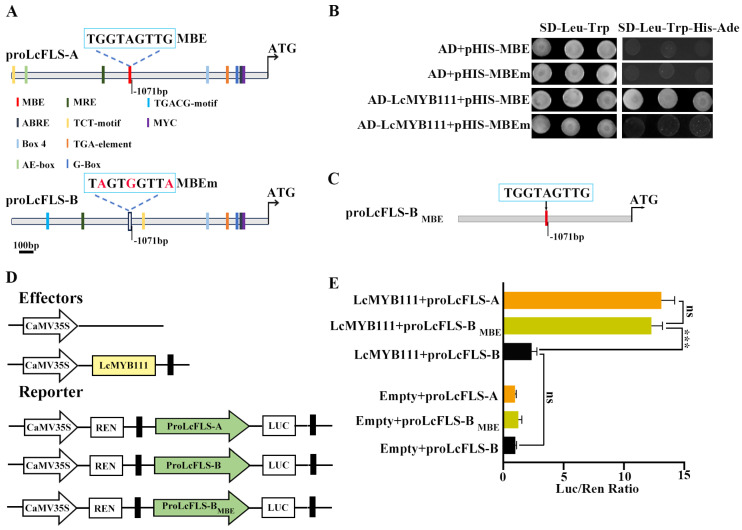
LcMYB111 regulates *LcFLS* gene expression by directly binding to specific MBE of proLcFLS. (**A**) Schematic diagram showing the predicted promoter regulatory elements of proLcFLS. Light regulatory elements MRE, ABRE, Box4, TCT-motif, HY5 binding element G-box, and transcription factor binding element MYC are marked with varied colors. Among them, the MYB-binding element of MYB111 (MBE) is marked with red, and three mutant bases of MBEm are marked with red; HY5 binding element G-box is marked with light blue. (**B**) Interaction of LcMYB111 with the MBE and MBEm of proLcFLS in Y1H assays. The interaction was determined by the growth of the yeast cells co-transformed with the indicated combinations of the plasmids on a synthetic dropout medium lacking Leu and Trp (SD-Leu-Trp) and a synthetic dropout medium lacking Leu, Trp, His, and adenine (SD-SD-Leu-Trp-His-Ade). AD, pGADT7; pHis, pHIS2. (**C**) The three bases of proLcFLS-B mutation were reverted to MBE by site-directed mutagenesis. (**D**) Diagram of constructs used for dual-luciferase (LUC) reporter assay as shown in (**E**). (**E**) Transcription activity analysis showed that LcMYB111 regulates *LcFLS* expression relying on MBE. LUC/REN ratios represent the transcription regulation activities and were normalized to the activity of control. All the bars indicate the mean ± SD of three repeated experiments (ns: no significance, *** *p* < 0.001; one-way ANOVA with Tukey’s tests).

In *A. thaliana* and *Brassica campestris*, *MYB111* has been proven to promote flavonol biosynthesis by binding to specific cis-elements in promoters of *FLS* [49,50]. Therefore, PlantCARE was used to predict promoter regulatory elements of proLcFLS-A and proLcFLS-B, which revealed that both allelic promoters contained a large number of light regulatory elements: MRE, ABRE, Box4, TCT-motif, G-box (HY5 binding element), and MYC transcription factor binding element (Figure 4A). And most importantly, the specific MYB-binding element of MYB111 (MBE) reported in *A. thaliana* [50] was found in proLcFLS-A (TGGTAGTTG). Interestingly, the specific MYB-binding element of MYB111 has three bases of mutation (MBEm) in proLcFLS-B (TAGTGGTTA).

The results of yeast one-hybrid (Y1H) assay indicated that LcMYB111 is bound to the MBE of proLcFLS-A, whereas there was no interaction between LcMYB111 and the mutated cis-element (MBEm) (Figure 4B). Therefore, we used LUC assay (Figure 4D) to test whether the mutated cis-element affects the DNA-binding activity of LcMYB111. Through site-directed mutagenesis, the three bases of proLcFLS-B mutation were reverted to MBE (Figure 4C), and a new promoter was produced (proLcFLS-B_MBE_). LUC assay results indicated that LcMYB111 had obvious transcriptional activation activity with proLcFLS-A or reverse mutation of proLcFLS-B with MBE (proLcFLS-B_MBE_), while it showed no transcriptional activation activity with proLcFLS-B compared with the control (Figure 4E). These results indicated that LcMYB111 could positively regulate *LcFLS* expression by binding to the specific MBE (TGGTAGTTG).

### 2.5. LcHY5 Interacts with LcMYB111 and Binds to G-Box of ProLcFLS to Promote Flavonol Synthesis

**Figure 5:**
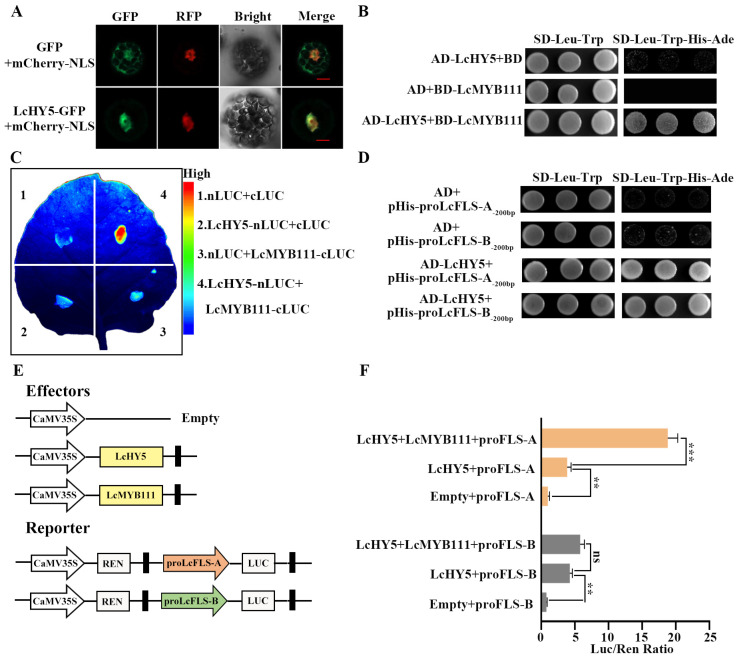
Physical interaction of LcHY5 with LcMYB111 and G-box of ProLcFLS. (**A**) LcHY5-GFP fusion constructs were used to determine the subcellular localization of LcHY5 in the protoplast cells isolated from *Arabidopsis* leaves. NLS-mCherry was used as a nuclear marker. Fluorescent images of GFP and mCherry were captured with a confocal laser scanning microscopy and are shown in green and red, respectively. Scale bars = 20 μm. (**B**) LcHY5 interacted with LcMYB111 in Y2H assays. Protein interaction was determined by the growth of the yeast cells co-transformed with the indicated combinations of the plasmids on a synthetic dropout medium lacking Leu and Trp (SD-Leu-Trp) and a synthetic dropout medium lacking Leu, Trp, His, and adenine (SD-Leu-Trp-His-Ade). AD, pGADT7; BD, pGBKT7. (**C**) LCI assay showing the interaction between LcHY5 and LcMYB111 in the leaves of *Nicotiana benthamiana*. Left: a representative leaf image. Right: the colored scale bar indicates luminescence intensity. (**D**) Binding of LcHY5 to the promoters of proLcFLS-A and -B in Y1H assays. For the yeast one-hybrid assay, an empty pHIS2.1 vector was used as a negative control. Various combinations of different constructs were transformed into the yeast strain Y187, and the protein–DNA interaction was determined based on the growth of positive transformants on SD-Leu-Trp-His-Ade. (**E**) Diagram of constructs used for dual-luciferase (LUC) reporter assay in *Arabidopsis* protoplasts as shown in (**F**). LcHY5 and LcMYB111 were used as effectors. (**F**) Transcription activity analysis showing the effect of LcHY5 and LcMYB111 on the transactivation of proLcFLS. LUC/REN ratios represent the transcription regulation activities and were normalized to the activity of control. The error bar represents the standard deviation of three biological replicates. No significance (ns), asterisk represents the statistical significance (** *p* < 0.01; *** *p* < 0.001).

LcMYB111 could regulate the synthesis of flavonols by directly binding to the promoters of *LcFLS-A*. Also, various studies found that HY5 interacts with MYB12 to promote flavonol biosynthesis [51,52,53], while the regulatory roles of these components are yet to be investigated in litchi. Therefore, we used the amino acid sequence of AtHY5 to blast against the litchi genome database, and we found a homologous gene with the highest score, which is named *LcHY5*. Then, we cloned and sequenced the open reading frames (ORFs) of *LcHY5* from the representative EEM cultivar ‘SYH’ and MLM cultivar ‘NMC’, respectively, and found that both cultivars have the same amino acid sequence of LcHY5 which shared 79.8% similarity with AtHY5 (Figure S6). The full-length coding sequence of the LcHY5 was fused in frame to the N-terminus of Green Fluorescent Protein (GFP) driven by the CaMV35S promoter. Then, the resulting fusions were co-expressed with the nuclear marker NLS-mCherry in the *Arabidopsis* protoplasts. Confocal microscopic analysis showed that GFP localized in both cytoplasm and nucleus, while the fluorescent signal of LcHY5-GFP was co-localized with NLS-mCherry in the nucleus, suggesting its function as a TF (Figure 5A).

Yeast two-hybrid (Y2H) assay was performed to detect the interactions between LcHY5 and LcMYB111. The full-length ORF of LcHY5 was fused to GAL4 activation domain (AD-LcHY5), and that of LcMYB111 was fused to DNA-binding domain (BD-LcMYB111) vectors. The AD-LcHY5 was co-transformed with the BD-LcMYB111 into yeast cells AH109, and the interaction of them was evaluated based on the growth of yeast cells on SD-Trp-Leu-His-Ade (SD-4) medium plates. As shown in Figure 5B, the yeast cells harboring AD-LcHY5 and BD-LcMYB111 pairs could well grow on the SD-4 selective medium, suggesting that these LcHY5 proteins directly interacted with LcMYB111 in yeast. Moreover, we performed a firefly luciferase complementation imaging (LCI) assay, and a strong fluorescent signal appeared after co-expression of LcHY5-nLUC/cLUC-LcMYB111 constructs, while no obvious fluorescent signal was detected when neither the LcMYB111-nLUC, cLUC-LcMYB111, nor nLUC/cLUC constructs were co-expressed in *Nicotiana benthamiana* leaf cells (Figure 5C), which demonstrated that interaction between LcMYB111 and LcHY5 had occurred.

Previous study found that HY5 can activate the *FLS* promoter to increase the flavonoid content [54]. As a HY5 binding site G-box was found at −168 bp of proLcFLS-A and proLcFLS-B through the abovementioned promoter regulatory elements analysis, 200 bp sequence upstream of start codon ATG of LcFLS-A and-B, proLcFLS-A_-200bp_ and proLcFLS-B_-200bp_ were co-transferred to yeast with LcHY5, and Y1H assays revealed that LcHY5 could bind to the promoters of *LcFLS-A* and *LcFLSA-B* (Figure 5D). We further investigated the regulatory function of LcHY5 on proLcFLS-A and proLcFLSA-B with the LUC assay and constructs schematically demonstrated in Figure 5E in *Arabidopsis* protoplasts. The results showed that proLcFLS-A and proLcFLSA-B were strongly activated when infiltrated with LcHY5; however, no significant differences appeared between the allelic promoters (3.0- and 2.4-fold). The surprise is that the activation was increased when both allelic promoters were co-infiltrated with LcMYB111 and LcHY5, indicating a possible additive effect of LcHY5 and LcMYB111. However, the activation of proLcFLS-A was significantly increased from 3.9- to 18.8-fold, while the activation of proLcFLS-B was slightly increased from 4.2- to 5.78-fold, when LcMYB111 was co-infiltrated with LcHY5 (Figure 5F). This discrepancy may be due to the fact that LcMYB111 has higher transcriptional activation with proFLS-A compared to proFLS-B.

## 3. Discussion

Flavonol synthase is a crucial enzyme in flavonol biosynthesis, and flavonols are pivotal regulators of flower color [12,17]. Two different alleles of *LcFLS, LcFLS-A* and *LcFLS-B*, have been identified in litchi cultivars in our previous study, with EEM cultivars only harboring *LcFLS-A* allele, while MLM cultivars only harbor *LcFLS-B* allele [48]. In the present work, both LcFLS-A and -B proteins were confirmed to exhibit FLS activity which could convert dihydroflavonols (DHQ and DHK) to flavonols (quercetin and kaempferol) (Figure S1A,B). We further successfully expressed *LcFLS-A* and *LcFLS-B* in tobacco; the flower color of *OE-LcFLS-A* and *OE-LcFLS-B* transgenic tobacco was lighter and almost white compared with the pink WT tobacco (Figure 1B). The *FLS* genes from *R. rugosa*, *P. persica*, or *P. hybrida* were expressed in tobacco, and showed the similar phenotypes [13]. Meanwhile, the resulting flowers contained increased levels of flavonols (kaempferol and quercetin) and decreased levels of anthocyanin (Figure 1C). Alike, expressing *ZmFLS1*, *OsFLS*, and *AcFLS-HRB* in tobacco increased flavonol levels and decreased anthocyanin levels of flowers [55,56]. These inverse correlations between flavonol and anthocyanin production are commonly observed in the *FLS* transgenic plants [7,10,57] due to the competition between FLS and DFR for dihydroflavonols, which creates a critical branch point separating the flavonol and anthocyanin biosynthetic pathways. Our results confirm that both *LcFLS-A* and *LcFLS-B* are functional *FLS* genes and indicate that the functionality of FLS is well conserved in plants. However, phenotypes or flavonol levels of *OE-LcFLS-A* and *OE-LcFLS-B* transgenic tobacco flowers show no significant difference; therefore, the two *LcFLS* alleles of EEM and MLM cultivars may have semblable function.

Flavonol synthesis is strictly regulated at the transcriptional level by several tissue-specifically expressed R2R3-MYB transcription factors [31,32,33,34]. *Arabidopsis* AtMYB11, 12, and 111 of SG7 were transcriptional activators [26,58]. Meanwhile, R2R3-MYB repressors, such as *Arabidopsis* AtMYB7 and AtMYB4 of SG4, have been demonstrated to be regulators of flavonol biosynthesis in plants [40]. In this study, we searched for homologous R2R3-MYB genes in the litchi genome using sequences of SG4 and SG7 as queries. We obtained four homologous genes, among which LcMYB111 clustered with SG7, while LcMYB6, LcMYB308, and LcMYB 330 clustered with SG4 (Figure 2A), suggesting their positive and negative functions in regulating flavonol biosynthesis, respectively. The ORF sequences of the four LcMYBs cloned from EEM and MLM cultivars were strictly conserved, with the protein sequence of LcMYB111 and LcMYB330 of both cultivars being identical, and LcMYB6 and LcMYB308 of both cultivars shared 98% and 97% identity, respectively (Figure 2B and Figure S3). Nonetheless, two allelic promoters of *LcFLS* were obtained, with EEM cultivars only harboring proLcFLS-A, while MLM cultivars only harbor proLcFLS-B (Figure 2C). Further, LUC assay confirmed that *LcMYB6*, *LcMYB308*, and *LcMYB330* function as negative regulators on proLcFLS-A and -B (Figure 3B); however, there was no significant difference in the degree of transcriptional repression between the two allelic promoters. Interestingly, LUC assay results showed that LcMYB111 had obvious higher transcriptional activation effect with proLcFLS-A than proLcFLS-B (9-fold vs. 2.5-fold) (Figure 3A,B). In addition, expression analysis also showed that the transcript levels of *LcFLS* and *LcMYB111* in mature leaves and flower buds were generally significantly higher in EEM cultivars compared with MLM cultivars (Figure 3C), while there was no general difference of the expression pattern of the *LcMYB6*, *LcMYB308*, and *LcMYB330* between EEM and MLM cultivars (Figure S5). Therefore, LcMYB111 may be responsible for the higher expression of *LcFLS* in EEM cultivars than MLM cultivars.

AtMYB111 and BcMYB111 have been proven to promote flavonol biosynthesis by interacting with specific cis-elements in the promoters of FLS (TGGTAGTTG) [39,49]. To explore the reason for the difference of transcriptional activation ability of LcMYB111 with two allelic promoters of *LcFLS*, promoter regulatory elements analysis was further performed, which showed that the specific MYB-binding element of MYB111 (MBE) was found in proLcFLS-A, while there is a mutation of three bases of MBE (MBEm) in ProLcFLS-B (Figure 4A). Y1H assay indicated that LcMYB111 interacted with the MBE sequences of *LcFLS*, while it showed no interaction with mutated cis-element MBEm (Figure 4B). Site-directed mutagenesis and LUC assay further confirmed that LcMYB111 could positively regulate *LcFLS* expression by directly binding to the specific MBE (Figure 4C–E).

**Figure 6:**
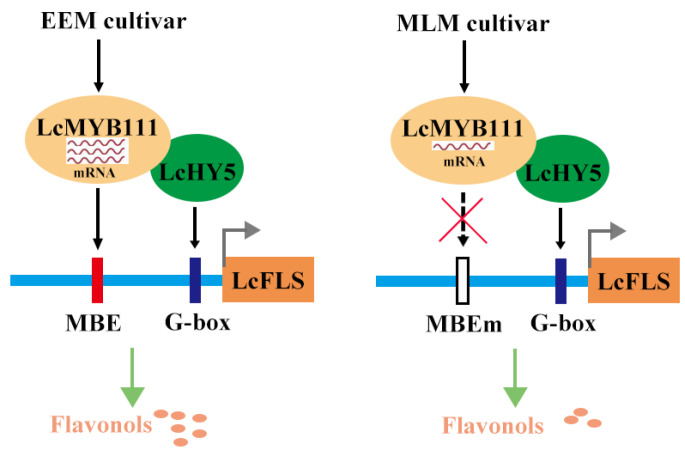
A proposed working model of LcMYB111 and LcHY5 in flavonol accumulation in EEM and MLM cultivars. The expression levels of *LcMYB111* in EEM cultivar is much higher than those in MLM cultivar. Furthermore, LcMYB111 specifically activates proLcFLS of EEM cultivar with MBE, while it is unable to activate proLcFLS-B of MLM cultivar with mutated MBE (MBEm). LcHY5 promotes *LcFLS* gene expression by directly binding to the G-box of the *LcFLS* promoter, and LcHY5 also interacts with LcMYB111 to enhance the activation of *proLcFLS*. Therefore, transcription levels of *LcFLS* in EEM cultivar are higher than those of MLM cultivar. The red wavy line indicatesthe expression abundance of LcMYB111, the red rectangles indicate MBE sequences, the purple rectangles indicate G-box sequences, and the long blue strip indicates the *LcFLS* promoter.

Recent studies also showed that HY5 is a positive regulator of anthocyanin accumulation through direct binding to the promoters of genes involved in anthocyanin biosynthesis [46,59]. HY5 directly binds to the G-box motifs in the promoters of anthocyanin biosynthesis genes, such as *CHS*, *CHI*, *FLS*, *MYB111*, and *MYB12*, thereby regulating anthocyanin biosynthesis in *Arabidopsis* [44]. MdHY5, PyHY5, and AtHY5 recognize and bind G-box elements in the promoters of MdMYB10, PyMYB10, and AtMYB111 [43,46,47], enhancing their expression, and promote the accumulation of anthocyanins. In this study, we obtained the homologous LcHY5 in litchi which localized in the nucleus (Figure 5A). We performed Y2H, LCI, and Y1H assays, by which the interaction between LcHY5 and LcMYB111 (Figure 5B,C), as well as LcHY5 directly binding to the promoter of *LcFLS*, were verified (Figure 5D). Furthermore, LUC assay demonstrated LcHY5 could interact with LcMYB111 to promote *LcFLS* expression; interestingly, the transcription activation activity was higher with proLcFLS-A of EEM cultivars than proLcFLS-B of MLM cultivars (Figure 5E,F). Based on the above results, a working model of LcMYB111 and LcHY5 in flavonol accumulation in EEM and MLM cultivars was proposed (Figure 6).

In summary, we have confirmed that both *LcFLS-A* and *-B* alleles are functional *FLS* genes, transgenic *LcFLS* increased flavonol contents and then led to flower colors becoming lighter. *LcMYB6*, *LcMYB308*, and *LcMYB330* function as negative regulators of *LcFLS*. LcMYB111 could positively regulate *LcFLS* expression by directly binding to the MBE. LcHY5 and LcMYB111 can interact to enhance the activation of *proLcFLS*. The transcription levels of *LcFLS* in EEM cultivar were well above those in MLM cultivar, maybe due to the higher transcript levels of *LcMYB111* and LcMYB111 specifically activating proLcFLS of EEM cultivar. However, we still do not know whether other genes or other regulatory mechanisms regulate transcription levels of the *LcFLS* gene. Further research is needed in this area.

## 4. Materials and Methods

### 4.1. Plant Materials

The litchi representative EEM cultivars ‘Hemaoli’ (‘HML’), ‘Sanyuehong’ (‘SYH’) and MLM cultivars ‘Xianjinfeng’ (‘XJF’), ‘Guiwei’ (‘GW’), and ‘Nuomici’ (NMC) grown in the experimental orchard in the Institute of Fruit Tree Research, Guangdong Academy of Agricultural Sciences (Guangzhou, China), were used in this study. *Arabidopsis* (*A. thaliana*) ecotype Columbia-0 (Col-0) was grown in a 22 °C plant growth chamber with a 16 h light/8 h dark cycle (120 µmol photons m^−2^ s ^−1^). Wild-type K326 tobacco, transgenic tobacco, and *Nicotiana benthamiana* plants were grown in a 25 °C plant growth chamber with a 16 h light/8 h dark cycle (120 µmol photons m^−2^ s^−1^).

### 4.2. Expression and Purification of Recombinant LcFLS Protein in E. coli and Activity Assay

To construct the pET32a-LcFLS prokaryotic expression vectors, the coding sequences (CDS) of *LcFLS-A* and *-B* were amplified with a sequence-specific primer set (Table S1) and digested with *SacI* and *HindIII* and then inserted into the vector pET32a (linearized with *SacI* and *HindIII*). The pET32a-LcFLS vectors were transformed into *E. coli* strain BL12 (DE3) (Novagen, Darmstadt, Germany). Protein expression was induced at 24 °C for 6 h in the presence of isopropyl β-D-1-thiogalactopyranoside (IPTG) (0.2 mM) in 50 mL of LB medium. Bacterial cells were then harvested and sonicated in 2 mL of lysis buffer (50 mM of sodium phosphate, 150 mM of NaCl, 1% triton X-100 (*v*/*v*), 10% glycerol (*v*/*v*), 2 mM of dithiothreitol, and 1 mM of phenylmethylsulfonyl fluoride). After centrifugation (12,000× *g*, 4 °C, 8 min), 100 μL of Ni-NTA beads (Solarbio, Beijing, China) was added to the soluble bacterial lysate and incubated at 4 °C for 2 h with gentle rotation. The beads were collected, washed four times with 1× PBS (137 mM of NaCl, 2.7 mM of KCl, 100 mM of Na_2_HPO_4_, and 2 mM of K_2_HPO_4_, pH 7.4), and then eluted three times with one bead volume of elution buffer (50 mM of Tris-Cl, pH 8.0) and 10 mM of reduced glutathione. The purified recombinant proteins were verified by SDS−PAGE. The enzymatic activities of Trx-LcFLS were assayed in a 250 μL reaction containing 10 mM of α-ketoglutaric acid (disodium salt), 10 mM of ascorbic acid, 0.25 mM of ferrous sulfate, 100 mM of Na_2_HPO_4_ (pH 6.8), and 400 μM of dihydroquercetin or 1 mM of dihydrokaempferol as a substrate. Reactions were initiated by the addition of substrate and incubated at 30 °C for 1 h; then, 100 μL of ethyl acetate was added to the reactions and mixed vigorously for 1 min. After centrifugation, 50 μL of ethyl acetate layers was evaporated, and the residues were dissolved in 100 μL of methanol for HPLC analysis. HPLC analysis was performed with an InfinityLab Poroshell HPH-C18 (150 mm × 4.6 mm, 2.7μm; Agilent, Santa Clara, CA, USA) using an Agilent 1290 Infinity II system. Methanol and double-distilled water were used as mobile phases A and B, respectively. The linear elution gradient method was used as follows: 0–5 min, 80% B; 5–30 min, 80% B; 30–40 min, 0% B. The flow rate was maintained at 1.0 mL/min. An injection volume of 10 mL and a wavelength of 289 nm and 368 nm were used for analysis of dihydroflavonols and flavonols, respectively.

### 4.3. Transformation of Tobacco Plants

The coding sequences (CDSs) of *LcFLS-A* and *-B* were cloned and inserted into BsaI/Eco31I-digested pBWA(V)HS vector (Biorun, China) to create pBWA(V)HS-LcFLS-A and pBWA(V)HS-LcFLS-B plasmid. Tobacco genetic transformation was performed by transforming leaf discs with *Agrobacterium tumefaciens* GV3101 containing the pBWA(V)HS-LcFLS-A or pBWA(V)HS-LcFLS-B plasmid. HYG-F/R was used for positive identification of transgenic tobacco. The primers used are listed in Table S1. The specific transgenic methods for tobacco were performed as previously described [17]. Briefly, the K326 tobacco leaf discs were dissected and submerged in the *Agrobacterium* mixture. After two days of co-culture, the leaves were transferred to the medium for callus induction for about 10 days. The explants were cultured on a shoot-inducing medium and root-inducing medium containing 50 mg·L^−1^ hygromycin to select transgenic calli. Then, hygromycin-resistant plants were transplanted into a greenhouse. We obtained more than 20 T_0_ transgenic lines.

### 4.4. HPLC Analysis of Flavonols and Anthocyanins in Tobacco Petals

The accumulation of flavonols and anthocyanins in the petals of WT and *LcFLS*-*A* and *-B* overexpressing transgenic tobacco lines was analyzed as described previously [60]. Briefly, 100 mg of petal sample was ground in liquid nitrogen, 4 mL of 1% hydrochloric acid-methanol solution was added, vortexed for 1 min, and sonicated in an ice water bath for 30 min. After centrifugation at 11,000× *g* for 10 min at 4 °C, the supernatant was collected, and the mixture was passed through 0.22 μm organic phase filter membrane. A 10 μL aliquot of the extract was used in HPLC analysis. HPLC analyses of flavonols were performed as described above. For anthocyanin determination, poroshell 120 SB-C18 reversed-phase chromatography column was used (150 mm × 2.1 mm, 2.7 µm; Agilent, USA). The mobile phase consisted of 0.1% formic acid (A) and acetonitrile containing 0.1% formic acid (B). Anthocyanins were identified using isocratic elution within 10 min and were quantified based on standard curves generated for authentic standards.

### 4.5. Identification and Molecular Cloning of Genes

Litchi R2R3 MYB and LcHY5 genes were identified by protein blast of 3 members of SG4 (AtMYB4, AtMYB7, and AtMYB32), 3 members of SG7 (AtMYB11, AtMYB12, and AtMYB111) and AtHY5 against the litchi genome database (http://www.sapindaceae.com/ (accessed on 1 January 2022)). The coding sequences of *LcMYBs* and *LcHY5* were amplified using litchi cDNA as template. The 1800 bp sequences of proLcFLS-A and -B from start codon ATG were cloned using litchi DNA as template. The primers used for gene cloning are listed in Table S1.

### 4.6. Phylogenetic Analysis, Sequence Analysis and Promoter Element Analysis

The phylogenetic tree was constructed using the neighbor-joining method in the MEGA-X software with 1000 bootstrap replicates. Multiple sequence alignments of the amino acid sequences were generated using DNAMAN 6.0 software. The cis-acting elements in the LcMYB111 promoter were predicted with PlantCARE (http://bioinformatics.psb.ugent.be/webtools/plantcare/html/ (accessed on 1 January 2022)).

### 4.7. RNA Isolation and RT-qPCR Analyses

Total RNA was extracted using the RNAprep Pure Plant Plus Kit (TIANGEN, Beijing, China). The RNA was then reverse-transcribed into cDNA using the Primescript RT reagent Kit with gDNA Eraser (Perfect Real Time) (TaKaRa, Beijing, China), according to the manufacturer’s instructions. TB Green Premix Ex Taq II (TaKaRa, China) was used for RT-qPCR analysis. The following PCR program on a CFX Connect real-time PCR detection system (Bio-Rad, Hercules, CA, USA) was used: 95 °C for 2 min, 40 cycles of 95 °C for 10 s, and 60 °C for 20 s, followed by a melting curve program. The litchi Actin gene was used as an internal control. Relative expression levels of genes were calculated using the 2^−ΔΔCT^ method [61]. The primers used for RT-qPCR are listed in Table S1.

### 4.8. GUS Stain Analysis

After the double-enzyme digestion by *XhoI* and *SpeI*, proLcFLS-A and -B were connected to the pART27-GUS plasmid, and then the recombinant plasmid was transformed into agrobacterium and infiltrated into *N. benthamiana* leaves. The primers used are listed in Table S1. After three days, GUS stain analysis was performed using the GUS staining kit (Solarbio, China).

### 4.9. Transcriptional Activation Assay

The transcriptional activation activity was measured using a dual-luciferase reporter assay in *Arabidopsis* protoplasts [62]. ProLcFLS-A, ProLcFLS-B, and ProLcFLS-B_MBE_ were amplified and inserted into pGreenII 0800-LUC vector which were digested with *KpnI* and *BamHI* to generate the reporter construct. The coding sequences of LcMYB111, LcMYB6, LcMYB308, LcMYB330, and LcHY5 were amplified and inserted into the pGreenII 62-SK vector which were digested with BamHI and HidIII to generate 35S::LcMYB111 and 35S::LcHY5 as effector plasmids. All primers used are listed in Table S1. The indicated constructs were transfected into *N. benthamiana* leaves. After 3 days, the firefly and Renilla luciferase activities were measured using a Dual-Luciferase Reporter Assay System kit (Promega, Shanghai, China) in a 96-microplate luminometer instrument.

### 4.10. Y1H and Y2H Assay

The YIH assay was carried out according to the manufacturer’s instructions (TaKaRa, China). The MBE sequence (TGGTAGTTG) and MBEm sequence (TAGTGGTTA), which added 15 bp homologous sequences of upstream and downstream of pHIS2 vector, respectively, were synthesized (Sangon Biotech, Shanghai, China). The synthetic sequences were mixed at a volume ratio of 1 to 1 at 98 °C for 3 min and slowed down to room temperature for base-pairing renaturation; and then, the paired sequences were inserted into pHIS2 vector (digested with *EcoRI* and *SacI*) to generate pHIS-MBE and pHIS-MBEm by homologous recombination following the manufacturer’s instructions (Vazyme, Nanjing, China). Next, 200 bp sequence upstream of start codon ATG of proLcFLS-A and -B (proLcFLS-A_-200bp_ and proLcFLS-B_-200bp_) which include G-box (CACGTC) were inserted into *EcoRI*/*SacI*-digested pHIS2 vector by homologous recombination. SD-Leu-Trp and SD-Leu-Trp-Ade-His solid medium were used to examine the DNA–protein interaction. The Y2H assay was also carried out following the manufacturer’s instructions (TaKaRa, China). The cDNA of LcMYB111 was cloned into pGBKT7 (digested with *EcoRI*) as bait, and the cDNA of the LcHY5 was cloned into pGADT7 (digested with *BamHI*) as prey by homologous recombination following the manufacturer’s instructions. The primers used for cloning are listed in Table S1. Different pairs of bait and prey vectors were co-transformed into the yeast reporter strain AH109. Then, the different yeast transformants were grown on synthetic double drop-out SD/-Leu/-Trp (SD-2) and synthetic quartette drop-out SD/-Leu/-Trp/-Ade/-His (SD-4) solid medium to examine the protein–protein interaction.

### 4.11. Subcellular Localization

The cDNA of the LcHY5 were cloned into pCAMBIA1300 (linearized with *SmaI*) vector to in-frame fuse with a GFP gene (LcHY5-GFP) by homologous recombination, while *EcoRI*/*BamHI*-digested pBI221 vector was fused with an NLS-mCherry tag (NLS-mCherry). The primers used in this study are listed in Table S1. The plasmid combinations of LcHY5-GFP and nuclear marker NLS-mCherry were introduced into *Arabidopsis* protoplasts to determine their subcellular localization. The transfected protoplast cells were incubated in 1 mL of W5 solution (154 mM of NaCl, 125 mM of CaCl_2_, 5 mM of KCl, and 2 mM of MES at pH 5.7) supplemented with 10 mM of sucrose for 12 h in the dark at 22 °C before imaging with a laser scanning confocal microscope (LSM800, Zeiss, Jena, Germany).

### 4.12. Firefly LCI Assay

The coding sequences of LcMYB111 and LcHY5 were amplified and inserted into the EcoRI/BamHI-digested pCAMBIA1300-cLUC vector and EcoRI/KpnI-digested pCAMBIA1300-nLUC vector by homologous recombination, respectively [62]. All primers used are listed in Table S1. These plasmids were transformed into *Agrobacterium* strain GV3101 and infiltrated into 5-week-old *N. benthamiana* leaves. After three days of infiltration, the luminescence activity was captured with the Tanon-5200 chemiluminescent imaging system (Tanon, Shanghai, China).

### 4.13. Statistical Analysis

GraphPad Prism 9.0 and Microsoft Office Excel 2021 were used for statistical analysis. The significance of difference was examined by one-way ANOVA with post hoc Tukey’s tests using the SPSS Statistics program (version 25, IBM, NY, USA).

## Data Availability

Data are contained within the article or Supplementary Materials.

## Supplementary Materials

The supporting information can be downloaded at: https://www.mdpi.com/article/10.3390/ijms242316817/s1.
