# Supplementary material for: An LcMYB111-LcHY5 Module Differentially Activates an LcFLS Promoter in Different Litchi Cultivars
Source: Int J Mol Sci. 2023 Nov 27;24(23):16817. doi: 10.3390/ijms242316817 (PMC10706726; doi:10.3390/ijms242316817)
Supplement: Supplementary file 1 [file ijms-24-16817-s001.zip › ijms-2707690-supplementary/supplementary Figures.pdf]

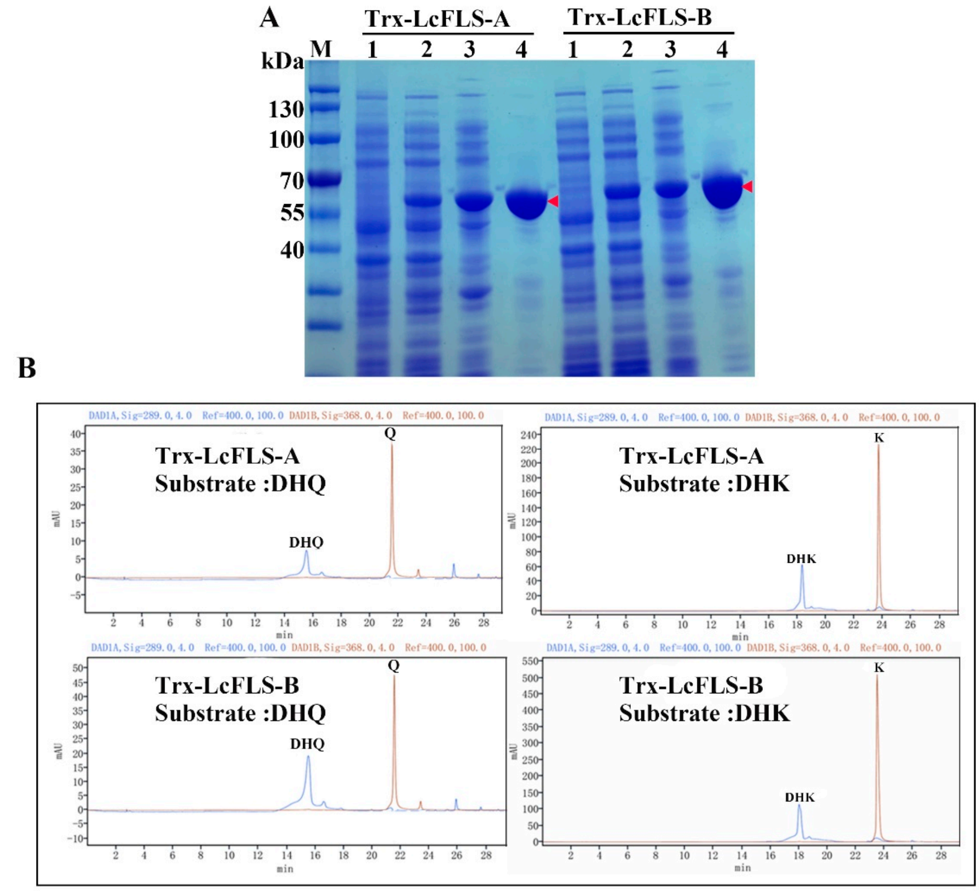

Figure S1. Protein purification and enzyme activity of recombinant LcFLS proteins. (A) SDS–PAGE analysis of the expression and purification of Trx-LcFLS-A and -B in *E. coli*. M, molecular weight size marker; 1, *E. coli* lysate before induction; 2, *E. coli* lysate after induction; 3, soluble fraction isolated after sonication of induced cells; 4, purified recombinant Trxa-LcFLSs. The molecular weights of Trx-LcFLS-A and Trx-LcFLS-B are approximately 58 kDa (LcFLS, 38 kDa ; Trx, 20 kDa), and the red triangle indicates that the recombinant proteins in SDS–PAGE. (B) HPLC chromatograms of products kaempferol (K) and quercetin (Q) from the in vitro reactions of Trx-LcFLS-A and Trx-LcFLS-B with dihydrokaempferol (DHK) and dihydroquercetin (DHQ) as substrates. Dihydroflavonols and flavonols were detected at 289 nm and 368 nm.

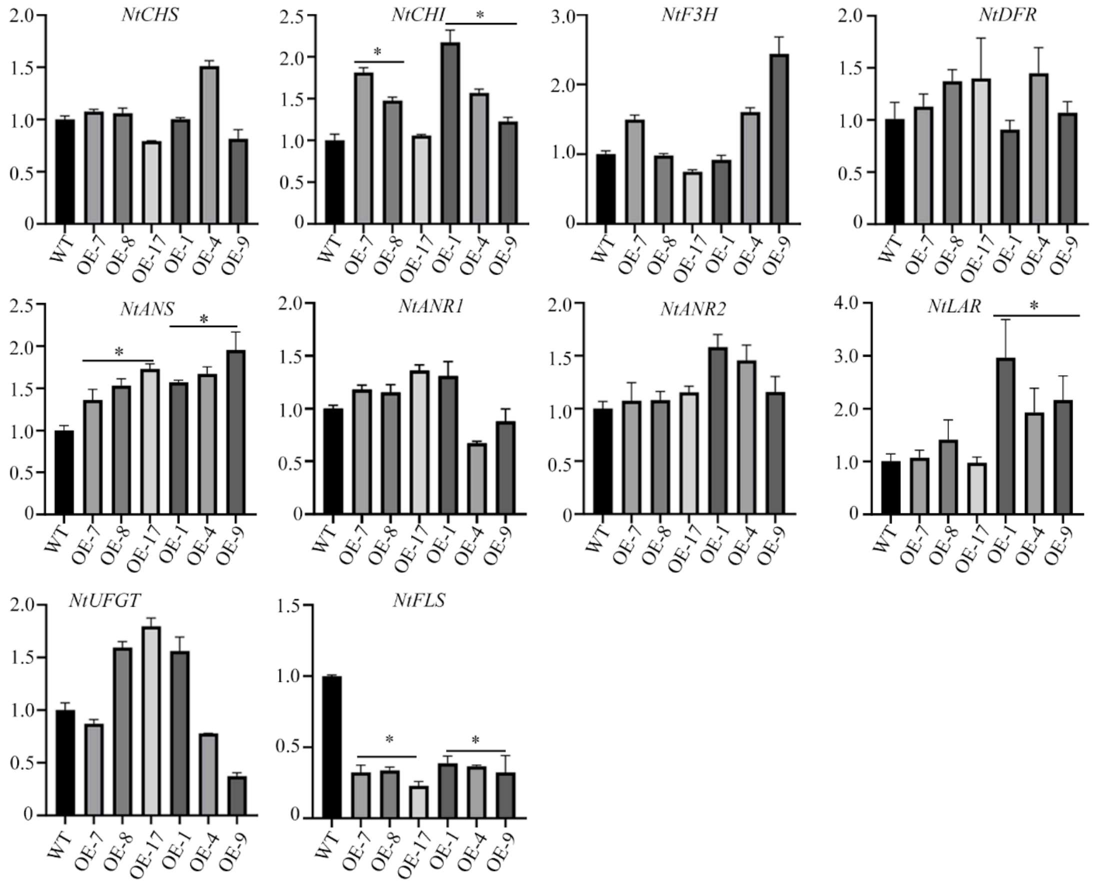

Figure S2. The expression levels of flavonoid pathway genes in WT and LcFLS-A and-B overexpressing transgenic lines. The nucleotide sequences of flavonoid biosynthesis pathway enzymes were download from the Sol Genomics Network (https://solgenomics.com/tools/blast/): NtCHS, chalcone synthase; NtCHI, chalcone isomerase; NtF3H, flavanone 3-hydroxylase; NtDFR, dihydroflavonol 4-reductase; NtANS, anthocyanidin synthase; NtANR, Anthocyanidin reductas; NtLAR: Leucoan-thocyanidin reductase; NtUFGT, UDP-glucose: flavonoid-3-O-glucosyltransferase and NtFLS, flavonol synthase; normalized against NtACTIN. The mean ±SD from three independent experiments were shown. Statistical significance was determined by one-way ANOVA with Tukey’s tests. Asterisks indicated significantly expression difference (*p< 0.05).

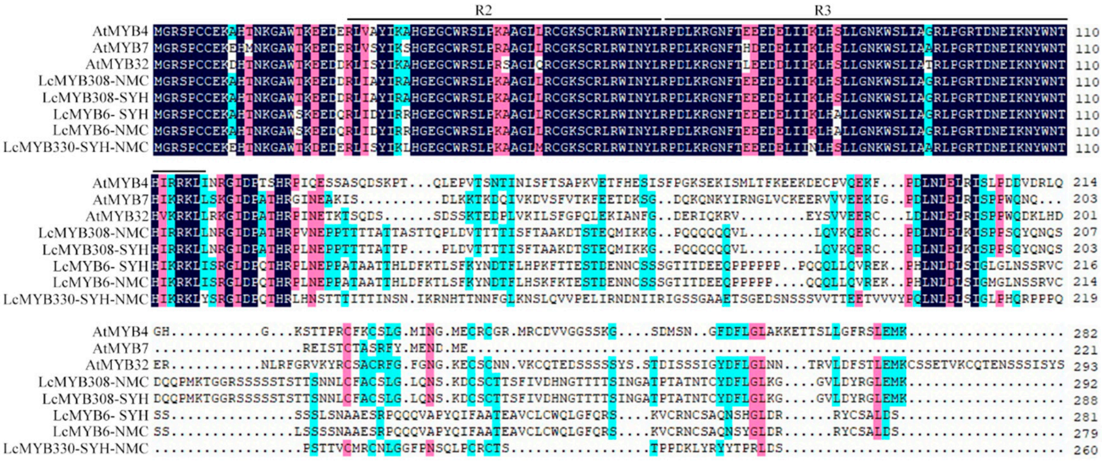

Figure S3. Sequence alignment of LcMYB6, LcMYB308 and LcMYB330 with flavonol regulators AtMYB4, AtMYB7, and AtMYB32. The alignment was generated using the DNAman program.The R2 and R3 repeats of the MYB domain are marked with blacklines.

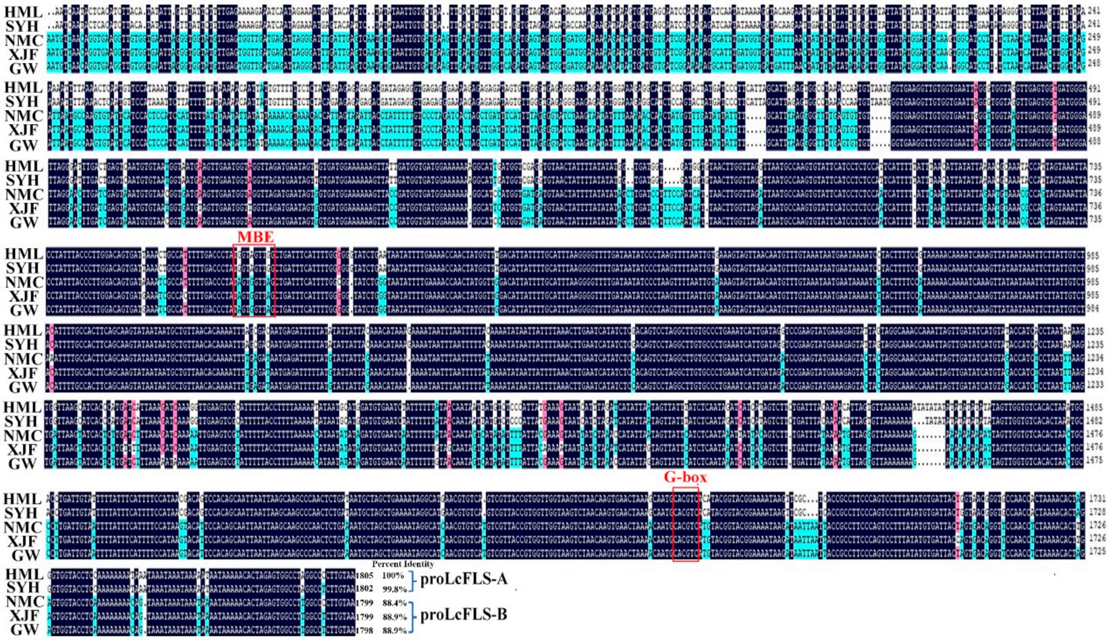

Figure S4. Sequence alignment of proLcFLS. The 1.8 kb promoter sequences of *LcFLS* were cloned from representative EEM cultivars ('SYH' and 'HML ') and MLM cultivars ('NMC', 'XJF' and 'GW ') respectively. *LcFLS* promoters (*proLcFLS*) also have two allelic sequences, which were named as proLcFLS-A and proLcFLS-B, with EEM cultivars only harbour proLcFLS-A while MLM cultivars only harbour proLcFLS-B. Red frame indicate MYB-binding element of MYB111 (MBE) and G-BOX (HY5 binding site). The alignment was generated using the DNAman program.

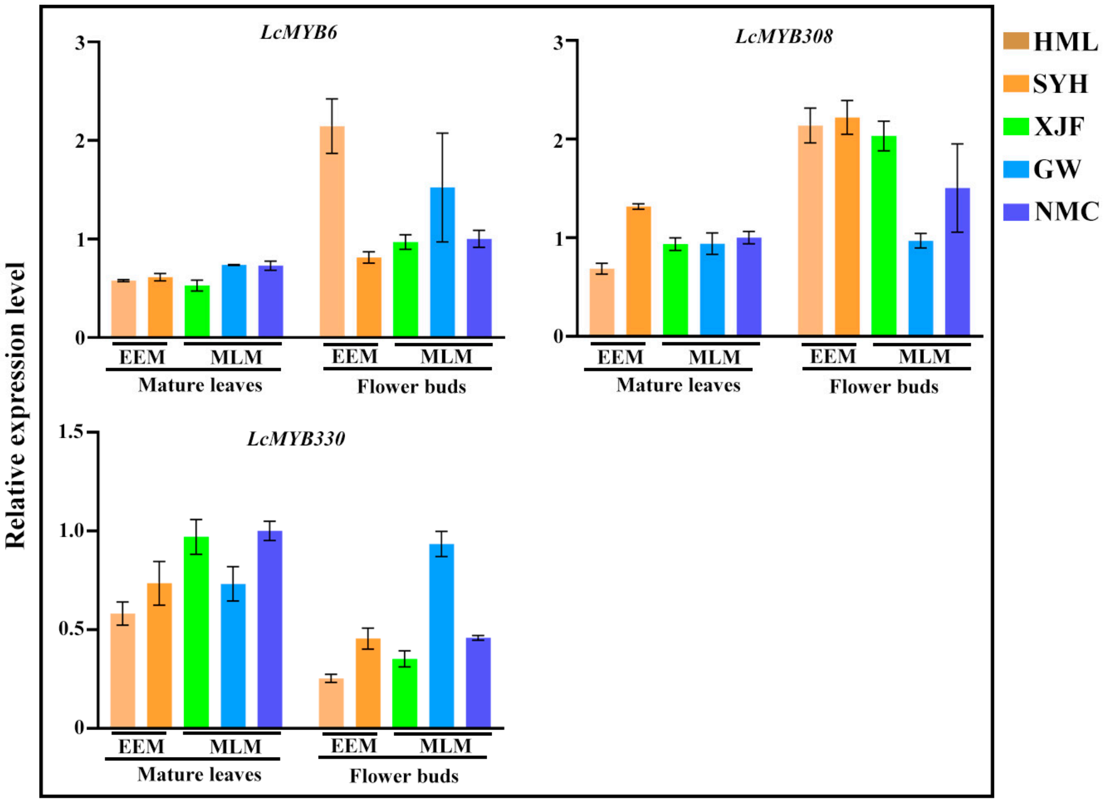

Figure S5. Expression pattern of *LcMYB6*, *LcMYB303* and *LcMYB111* in mature leaves and flower buds of litchi EEM cultivars ('HML' and 'SYH') and MLM cultivars ('XJF', 'GW' and 'NMC'). Relative gene expression levels were calculated as the ratio of 'HML', 'SYH', 'XJF', and 'GW' to 'NMC' respectively after being normalized against *LcActin*. The mean ±SD from three independent experiments were shown.

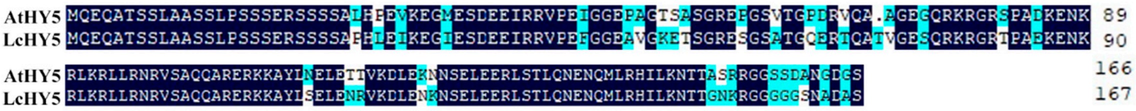

Figure S6. Sequence alignment of AtHY5 and LcHY5. LcHY5 which shared 79.8% similarity with AtHY5. The alignment was generated using the DNAman program.
